# Diabetes Mellitus in Kidney Transplant Recipients and New Hypoglycemic Agent Options

**DOI:** 10.3390/ijms26135952

**Published:** 2025-06-20

**Authors:** Giulia Bartoli, Andrea Dello Strologo, Maria Arena, Maria Josè Ceravolo, Anna Paola Mitterhofer, Francesco Pesce, Giuseppe Grandaliano

**Affiliations:** 1 Nephrology and Dialysis Unit, Policlinico Universitario Tor Vergata, 00133 Rome, Italy; giulia23bartoli@gmail.com (G.B.); mariajose.ceravolo@gmail.com (M.J.C.); annapaola.mitter@uniroma2.it (A.P.M.); 2 Department of Translational Medicine and Surgery, Università Cattolica del Sacro Cuore, 00168 Rome, Italy; andreadellostrologo@gmail.com (A.D.S.); giuseppe.grandaliano@policlinicogemelli.it (G.G.); 3 Department of Nephrology and Dialysis, Azienda USL Roma 6, 00040 Albano Laziale, Italy; 4 Nephrology, Dialysis and Transplantation Unit, Fondazione Policlinico Universitario A. Gemelli IRCCS, 00168 Rome, Italy; maria.arena@policlinicogemelli.it; 5 Department of Systems Medicine, University of Rome Tor Vergata, 00133 Rome, Italy; 6 Division of Renal Medicine, Ospedale Isola Tiberina—Gemelli Isola, 00186 Rome, Italy

**Keywords:** kidney transplantation, post-transplant diabetes mellitus, hypoglycemic agent, chronic kidney disease, sodium glucose co-transporter type 2 inhibitors, glucagon-like peptide-1 receptor agonists, nonsteroidal mineralocorticoid receptor antagonist

## Abstract

Diabetes mellitus (DM) is frequent in kidney transplant recipients (KTRs), reducing graft and patient survival. In recent years, hypoglycemic agents have been approved for chronic kidney disease (CKD) patients, such as sodium glucose co-transporter type 2 inhibitors (SGLT2is), glucagon-like peptide-1 receptor agonists (GLP1RAs), and nonsteroidal mineralocorticoid receptor antagonists (ns-MRAs), such as finerenone. Several studies demonstrated the ability of these drugs to reduce cardiovascular (CV) events and kidney disease progression in diabetic CKD patients. In this review, we will describe their use in KTRs with type 2 DM or post-transplant diabetes mellitus (PTDM), focusing on the potential positive effects. In particular, we will report literature data from observational studies, meta-analyses, and clinical trials. Based on their mechanism of actions, these drugs may balance the negative effects of immunosuppressive therapy on metabolic balance, reducing the risk of PTDM and CV events, that remain the first cause of death in KTRs. Generally, SGLT2is and GLP1RAs appear to be safe and efficacious in KTRs, and no interaction with immunosuppressive drugs or an increased risk of rejection has been reported. Regarding finerenone, no literature data are available and only one clinical trial is ongoing. In conclusion, although the 2022 KDIGO guidelines recommend caution in KTRs, the last meeting in Vienna on PTDM encourages their use in this population.

## 1. Introduction

Kidney transplantation (KT) is the best treatment for end-stage kidney disease (ESKD), improving patient survival and quality of life compared to dialysis [1,2]. On the other hand, long-term graft and patient outcomes may be worsened by several post-transplant complications, such as metabolic disorders, in particular diabetes mellitus (DM).

DM is one of the main complications in kidney transplant recipients (KTRs) [3]. In addition, diabetes mellitus (DM) is the main cause of chronic kidney disease (CKD) and end-stage kidney disease (ESKD) in the United States and worldwide [4].

In KTRs, DM is differentiated into pre-transplant DM, transient post-transplant hyperglycemia that occurs in the immediate-to-early post-transplant setting, new-onset diabetes mellitus after transplantation (NODAT) [5], and post-transplantation diabetes mellitus (PTDM) [6].

Transient post-transplant hyperglycemia is caused by post-surgical stress and high-dose glucocorticoids. This condition is very common (90% of recipient patients) and usually resolves within the first weeks after transplantation. However, it is a key risk indicator of PTDM [6]. NODAT is a term coined in 2003 by the international consensus guidelines to describe the development of DM after kidney transplantation due to alterations in glucose metabolism [5]. The group does not include patients with unrecognized diabetes prior to transplant. The term PTDM was adopted in 2014 after a consensus meeting in Vienna, in order to describe the presence of diabetes after transplantation and to include patients with undiagnosed diabetes during the pre-transplant period [6].

## 2. Prevalence and Diagnosis of PTDM

Despite its clinical relevance, PTDM has been underdiagnosed due to a lack of a standardized definition or diagnosis, and for this reason, its prevalence is not well defined [7,8]. However, based on its definition, PTDM diagnosis should be performed when patients are stable on their maintenance immunosuppression therapy (stable kidney allograft function, absence of acute infection or rejection) to avoid the inclusion of patients with transient hyperglycemia. Consequently, diagnosis should not be made in patients within the first six weeks after transplantation [6]. Diagnostic criteria for PTDM in KTRs are the same as in non-transplant patients and include an analysis with the oral glucose tolerance test (OGTT), hemoglobin A1c (HbA1c), and fasting glucose sample [9,10] (Table 1). For KT patients, the gold standard for PTDM diagnosis is the OGTT in that these patients often show postprandial hyperglycemia and normal fasting glucose levels. In addition, the OGTT detects the impaired glucose tolerance (IGT), which is a risk factor for the development of PTDM and an independent risk factor for cardiovascular (CV) disease and mortality [11]. Finally, HbA1C is not recommended for PTDM diagnosis during the first three months after transplantation [4].

## 3. Risk Factors for PTDM

**Figure 1:**
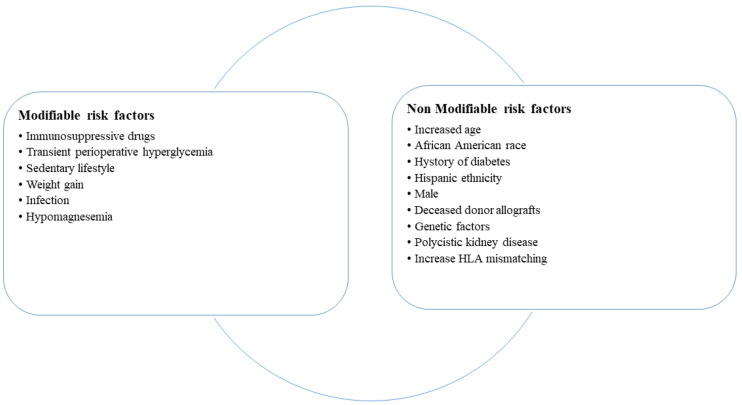
Risk factors for the development of post-transplant diabetes mellitus.

KTRs are at risk to develop PTDM or pre-diabetes because of several risk factors, which are differentiated into modifiable risk factors due to transplantation and non-modifiable risk factors due to recipient characteristics (Figure 1), such as increased age, male sex, race and ethnicity, and polycystic kidney disease. Among transplantation factors, an increased HLA mismatching (MM) and a deceased-donor allograft are associated with a high risk of PTDM. In the post-transplant period, immunosuppressive therapy, obesity, weight gain, sedentary lifestyle, and infections [12] may induce hyperglycemia and PTDM that negatively impact graft and patient survival, increasing all causes of mortality [13].

Immunosuppressive drugs show several adverse effects with an important impact on metabolic balance, and thus with an increased risk of development of diabetes, dyslipidemia, obesity, and hypertension [14]. These comorbidities are associated with CV events and reduce patient overall survival. Immunosuppressive drugs are characterized by different diabetogenic effects. For example, among calcineurin inhibitors (CNIs), tacrolimus shows a higher diabetogenic effect compared to cyclosporine [15], and, together with corticosteroids [16], has more impact on metabolic balance. The diabetogenic effect is caused by a significant dysregulation in insulin signaling and insulin secretion [17]. In particular, tacrolimus causes a pancreatic β-cell dysfunction with decreased insulin signaling and a decreased synthesis and release of insulin [18]. Mammalian target of rapamycin inhibitor (mTORi) presents different effects that may induce hyperglycemia and glucose intolerance such as a reduction in beta-cell mass, interference with insulin signal transduction, and upregulation of gluconeogenic genes in the liver [19]. Sirolimus is associated with peripheral insulin resistance and decreased pancreatic β-cell proliferation [20]. Belatacept, a fusion protein that selectively inhibits T-cell activation, has a lower diabetogenic effect compared to CNI, being an alternative in KTRs with low immunological risk [21]. Antiproliferative agents, mycophenolate mofetil (MMF) or azathioprine, are not characterized by glycemic risk. Despite these differences, there are no indication for modified immunosuppression therapy based on the risk or the development of DM [22].

## 4. Treatment for PTDM

In recent years, new drugs have been approved for CKD patients, principally in type 2 DM, in order to reduce CV events and kidney disease progression, such as sodium glucose co-transporter type 2 inhibitors (SGLT2is) [23,24], glucagon-like peptide-1 receptor agonists (GLP1RAs) [25,26], and nonsteroidal mineralocorticoid receptor antagonist (ns-MRA) [27,28].

Kidney Disease: Improving Global Outcomes (KDIGO) guidelines 2022 [29] recommend their use in diabetic CKD patients; in particular, the first-line drug therapy for DM type 2 patients is with metformin and SGLT2i. If the glycemic target is not reached or a high CV or kidney disease progression risk persists, GLP1RA and ns-MRA can be added.

Actually, KDIGO recommend caution in KTRs because of the possible infective risk. On the other hand, in the last international consensus on PTDM in Vienna 2022 [22], meeting participants, all experts in PTDM, agreed to also adopt these drugs in transplant patients with outpatient hyperglycemia, while insulin is preferred for post-transplant hyperglycemia.

In this review, we will describe the use of these new drugs in KTRs [30,31], focusing on the potential positive effects. Based on their mechanism of actions, these drugs may balance the negative effects of immunosuppressive therapy on metabolic balance, reducing the risk of PTDM, dyslipidemia, obesity, and hypertension [32].

In particular, we will report literature data from observational studies, meta-analyses, and clinical trials obtained by research on PubMed.gov and Clinicaltrial.gov. The search terms used were kidney transplantation, post-transplant diabetes mellitus, type 2 diabetes mellitus, hypoglycemic agent, chronic kidney disease, sodium glucose co-transporter type 2 inhibitors, glucagon-like peptide-1 receptor agonists, and nonsteroidal mineralocorticoid receptor antagonists.

## 5. Novel Hypoglycemic Agents

### 5.1. Sodium Glucose Co-Transporter Type 2 Inhibitors

SGLT2is are hypoglycemic agents, recently approved in CKD and/or heart failure (HF) patients due to their CV- and renal-protective effect in patients with and without type 2 DM [24,33,34].

Several clinical trials have investigated the protective effect of these drugs, such as empaglifozin, canaglifozin, dapaglifozin, and ertugliflozin (EMPA-KIDNEY, DAPA-CKD, CREDENCE) [24,33,34,35]. These studies showed a significant reduction in all-cause mortality, CV mortality, hospitalizations because of HF, the requirement of kidney replacement therapy (KRT), and acute kidney injury (AKI). In April 2021, dapaglifozin was the first SGLT2i approved by the FDA for CKD patients regardless of DM.

**Figure 2:**
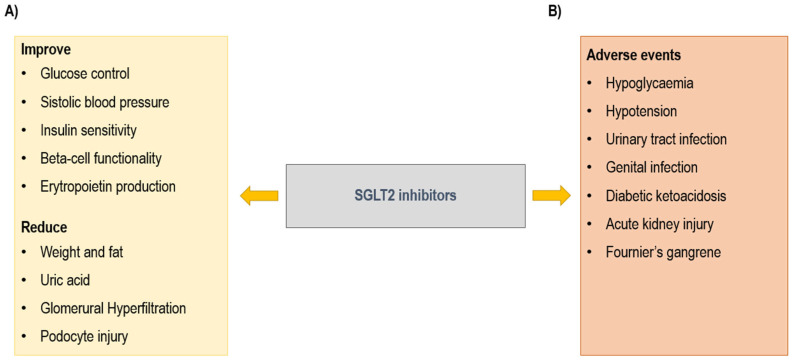
Sodium glucose co-transporter type 2 (SGLT2) inhibitors: (**A**) beneficial and (**B**) adverse effects.

SGLT2is reduce renal tubular glucose reabsorption, inducing a reduction in blood glucose without stimulation of insulin release. These drugs inhibit sodium-glucose co-transporter-2 (SGLT2) proteins, expressed in the proximal convoluted tubule of the kidneys, and responsible for 90% of filtered glucose reabsorption. Diabetic patients show an increased expression of SGLT2 in association with tubular growth [36]. The inhibition of SGLT2 co-transporter leads to a decreased reabsorption of sodium. In this way, SGLT2is induce glycosuria and natriuresis and consequently osmotic diuresis. Based on their mechanism of action, SGLT2is exert several positive effects that determine a nephroprotective effect and a reduction in CV events [37,38] (Figure 2). In particular, SGLT2is improve glucose control, with a reduction in HbA1c [37]; improve the control of systolic blood pressure, thanks to osmotic diuresis and natriuresis; induce weight loss; stimulate erythropoietin production; and improve beta cell functionality and insulin sensitivity. At the kidney level, SGLT2is reduce glomerular hyperfiltration and podocyte injury [37,38,39,40,41,42,43,44,45].

The most common adverse events (AEs) (Figure 2) are genital infections and urinary tract infections (UTIs) caused by glycosuria [46]. A serious genital infection is Fournier’s gangrene, which is a rare but potentially fatal event characterized by necrotizing fasciitis of the perineal soft tissues. Actually, 55 cases of Fournier’s gangrene have been identified over a period of 6 years [47]. Other AEs are hypoglycemia, principally when the drug is associated with insulin; hypotension; AKI; and diabetic ketoacidosis (DKA) [48].

As previously mentioned, DM is frequent in KTRs, reducing long-term graft and patient survival. As the main cause of death in KTRs is cardiovascular disease [32], the ability of SGLT2is to reduce CV events and kidney disease progression may be an important therapeutic weapon in these patients [49].

However, the AEs described above limit their use, principally due to infective risk [49]. Indeed, KT patients have an increased infectious risk compared to the non-transplant one because of abnormal genitourinary anatomy, which predisposes them to develop UTIs, as well as the maintenance immunosuppressive therapy [50]. UTIs are the most common infectious complications among KTRs. They may cause hospitalizations for sepsis with an increased risk of mortality, especially in the first months after transplantation. On the other hand, the use of SGLT2i may have several positive effects in that they reduce hyperfiltration and consequently proteinuria [51]. In addition, SGLT2is have an impact on metabolism and this is particularly useful in KTRs, which are characterized by dyslipidemia and alterations in lipid metabolism with an increased risk of CV events [52] due to long-term immunosuppressive drugs. Thanks to the reduction in glucose tubular reabsorption, SGLT2is decrease the metabolic demand and oxygen consumption, preserving tubular function and the Estimated Glomerular Filtration Rate (eGFR). Since SGLT2is improve the glycemic control, they induce a change from carbohydrate to lipid metabolism, with a reduction in fat and body weight. All these factors may reduce the risk of PTDM in KTRs, counterbalancing the effect of immunosuppressive agents.

SGLT2i treatment may also correct electrolyte anomalies, such as hyperuricemia, hypomagnesemia, and hyperkaliemia, and post-transplant anemia [40,41,42].

In addition, literature data also explore the effect of SGLT2i on gut microbiota, which is correlated with immune response, drug metabolism, and post-transplant complications in KTRs.

Based on the beneficial effects, several studies have investigated the use of SGLT2i in KT patients [49,53,54] (Table 2). Generally, these studies included patients carefully selected with stable renal functions and at many years after transplantation. By contrast, patients with recurring UTIs and in the immediate period after transplantation were generally excluded [55,56,57], in order to reduce the risk of infectious complications.

The first prospective placebo-controlled randomized clinical trial (RCT) was the EMPA-RenalTx [55], a single-center, prospective, controlled, double-blinded, randomized study that included 49 KTRs with PTDM after almost 1 year from transplantation. The study analyzed the efficacy and safety of empaglifozin vs. placebo, finding a reduction in HbA1c and body weight in the treated group. No significant differences in AEs, immunosuppressive drug levels, or graft function were observed.

Song et al. [58] described the use of SGLT2i in 50 KTRs with DM and eGFR > 30 mL/min (mean age, 57.03 ± 13.14 years; male, 66%), including patients in the first years of transplantation (median time of 319 days from transplantation to drug initiation). They excluded patients with AKI within 30 days or UTIs within 6 months. No differences in AEs, immunosuppressive drug levels, and eGFR decline were observed. Among EVs, no cases of DKA, amputations, or AKI episodes were reported. UTIs were observed in 14% of patients and 1 patient developed genital yeast infection.

Schwaiger et al. [57] analyzed the use of empaglifozin in 14 KTRs with PTDM (mean age, 56.5 ± 7.9 years; male, 50%; time after transplantation, 69.4 months), with the aim to interrupt insulin therapy. In the first 4 weeks, insulin therapy was stopped and an OGTT was performed at baseline and after 4 weeks of empaglifozin monotherapy. In empaglifozin-treated participants, oral glucose insulin sensitivity decreased and beta-cell glucose sensitivity increased at the 4-week and 12-month OGTTs. After 4 weeks, seven KTRs needed to reintroduce insulin in therapy. The study demonstrated that empaglifozin is safe in KTRs with PTDM but empaglifozin monotherapy is unable to reach a good glucose control. All patients completed the study and authors observed a reduction in body mass index, body weight, and waist circumference after 12 months.

In a Korean study, Lim et al. [59] described the effect of SGLT2i in 226 KTRs with DM in a multicenter study, confirming that SGLT2is improve the primary outcomes, with a decrease in all-cause mortality and death-censored graft failure and improved graft outcome. Regarding the expected eGFR dip during the first month, they found a correlation between the eGFR dip and time from transplantation and tacrolimus levels. A multicenter Spanish study [60] analyzed 339 diabetic KTRs (mean age, 61.6 ± 9.9 years; male, 73.7%; time after transplantation, 72.3 months) in SGLT2i treatment in order to define the safety profile of these drugs. As known, the most frequent AE was UTI (14% of patients) and the risk was higher in females and patients with a history of UTI.

Sheu et al. [61] analyzed international data from the TriNetX platform to compare diabetic KTR SGLT2i users (N, 1970) and non-users (N, 1970). The primary endpoints were all-cause mortality, major adverse cardiac events (MACEs), and major adverse kidney events (MAKEs) in diabetic KTRs. During a period of follow up of 3.4 years, authors demonstrated that SGLT2i users showed a significant reduction in risk of mortality (2.08% users vs. 9.54% non-users; aHR, 0.32), risk of MACE (4.44% vs. 13.87%; HR, 0.48), and risk of MAKE (8.93% vs. 22.54%; HR, 0.52). Regarding the safety profile, no significant differences in genitourinary infections were found in the two groups. This is the largest study investigating the use of SGLT2i in KTRs.

Literature data confirm the ability of SGLT2i to improve CV and graft outcomes with a safe profile also in diabetic KTRs [62]. However, all authors agree that controlled studies are needed. Actually, there are several ongoing RCTs (Table 3) with the aim to investigate the safety profile of these drugs and the effect of SGLT2i on metabolic risk and graft outcomes [63,64,65,66,67,68,69,70]. It is important to underline that almost all these trials excluded patients with a history of rejection, UTIs, and genital infections.

Other trials investigated the effect of SGLT2i on specific graft outcomes, such as kidney interstitial fibrosis, oxygen tension, and oxygen stress in kidney transplant patients [71,72,73].

**Table 3:** Clinical trials with SGLT2 inhibitors in kidney transplant recipients.

	Number of Patients	Time from Transplant	Intervention	Primary Endpoints	State
Effect of Adding Dapagliflozin to Allograft Dysfunction of Renal Transplanted Patients [69]	211 KTRs	1–5 years	Dapaglifozin 10 mg vs. placebo	Effect on renal function	Completed
Efficacy, Mechanisms and Safety of SGLT2 Inhibitors in Kidney Transplant Recipients (INFINITI2019) [64]	52 KTRs T2DM–PTDM	≥6 months	Dapaglifozin 10 mg vs. placebo	Reduced Blood pressure	Completed
Empagliflozin Treatment in Kidney Transplant Recipients (SEKTR) [66]	Veterans KTRs T2DM–PTDM	≥3 months	Empaglifozin 12.5 mg	Safety and efficacy	Recruiting
CardioRenal Effects of SGLT2 Inhibition in Kidney Transplant Recipients (CREST-KT) [63]	72 KTRs	12–60 months	Empaglifozin 10 mg vs. placebo	Safety and efficacy	Recruiting
The Efficacy, Mechanism & Safety of Sodium Glucose Co-Transporter-2 Inhibitor & Glucagon-Like Peptide 1 Receptor Agonist Combination Therapy in Kidney Transplant Recipients (HALLMARK) [68]	20 KTRs T2DM–PTDM	≥3 months	Dapaglifozin 10 mg plus semaglutide 1.0 mg/mL	Short-term efficacy and safety (12 weeks)	Recruiting
Can Dapagliflozin Preserve Structure and Function in Transplanted Kidneys? (DEAKTransplant) [71]	330 KTRs	≥6 weeks	Dapaglifozin 10 mg vs. placebo	Effect on eGFR slope; Effect on graft fibrosis and proteomic; Effect on metabolic risk	Recruiting
The RENAL LIFECYCLE Trial: A RCT to Assess the Effect of Dapagliflozin on Renal and Cardiovascular Outcomes in Patients With Severe CKD [67]	1500 severe CKD pts	Not specified	Dapaglifozin 10 mg vs. placebo	Reno- and cardio protective efficacy and safety	Recruiting
Effect of Empagliflozin vs Linagliptin on Glycemic Outcomes, Renal Outcomes & Body Composition in Renal Transplant Recipients With Diabetes Mellitus (EmLinaRenal) [70]	KTRs T2DM–PTDM	≥3 months	Empaglifozin 25 mg vs. linagliptin 5 mg	Effect on glycaemic outcomes; Effect on renal outcomes; Effect on body composition	Recruiting
Effects of Empagliflozin in Reducing Oxidative Stress After Kidney Transplantation [73]	40 KTRs T2 DM	Not specified	Empaglifozin 25 mg plus insulin vs. insulin	Effect on oxidative stress	Recruiting
SGLT2i in Diabetic Patients with Renal Transplantation [65]	72 KTRs PTDM	≥3 months	SGLT2i	Safety and efficacy	Active, not recruiting
Acute Effects of SGLT2 Inhibitor on Kidney Allograft Oxygen Tension (SGL-TX-MR) [72]	8 KTRs No DM	≥6 months	50 mg empaglifozin single dose vs. placebo	Change in kidney allograft cortical and medullary oxygen tension	Recruiting

KTRs = kidney transplant recipients; T2DM = type 2 diabetes mellitus; PTDM = post-transplant diabetes mellitus.

### 5.2. Glucagon-like Peptide-1 Receptor Agonists

GLP1RAs are hypoglycemic agents that improve glycemic control and weight loss. In recent years, several clinical trials demonstrated that GLP1RAs reduce the risk of major CV events and CKD progression in DM patients [25,74,75,76,77]. Subsequently, 2022 KDIGO guidelines recommended administering GLP1RAs as second-line treatment in patients with CKD, DM, and obesity [29]. Of particular note, the clinical trials that investigated their use in CKD patients have excluded KTRs.

GLP1RAs bind and activate GLP-1 receptors, determining an improvement in insulin sensitivity, a delay in gastric emptying, appetite suppression, and anti-atherogenic effects. Indeed, GLP1RAs mimic the incretin activity of GLP1, an endogenous hormone secreted after ingestion by small intestine cells with a role in insulin secretion by pancreatic beta cells. Incretin receptors, such as glucose-dependent insulinotropic polypeptide receptor (GIPR) and GLP1R, are also expressed in other organs, such as the liver, muscle, adipose tissue, central nervous system, heart, immune system, and kidneys [78]. The high expressions of GLP1R may explain the cardio- and renal-protective effects of GLP1RAs. Interestingly, GLP1RAs also present an anti-inflammatory effect [79] based on their direct effect on gut intraepithelial lymphocytes. Furthermore, GLP1RAs attenuate the induction of plasma tumor necrosis factor alpha (TNF-α) thanks to their binding to central neuronal GLP-1Rs.

Generally, GLP1RAs (i.e., dulaglutide, exenatide, semaglutide, lixisenatide) are well tolerated. The more frequent AEs are gastrointestinal (GI) symptoms, such as nausea, vomiting and diarrhea, pancreatitis, and cholelithiasis. The progressive increase in their use is associated with an increase in registered AEs. Other possible AEs are medullary thyroid cancer and diabetic retinopathy. Regarding adverse kidney events, the Food and Drug Administration adverse event reporting system (FAERS) described that AKI is the most common adverse kidney event, and that GLP1RAs may determine proteinuria [80]. AKI may be a consequence of GI effects, such as vomiting and diarrhea that cause hypovolemia and pre-renal AKI. Begun et al. [80] reported two cases of semaglutide-associated acute interstitial nephritis (AIN), which resolved after drug discontinuation and immunosuppressive therapy.

As regards the use of GLP1RAs in transplant patients, the principal concern is the potential interference with immunosuppressive drugs. Nevertheless, as GLP1RAs are eliminated by proteolytic degradation and degradation metabolites are eliminated through urine and feces [81], they do not interfere with the metabolism of immunosuppressive drugs, such as tacrolimus that is degraded by cytochrome P450. An alteration of immunosuppressive levels may be a consequence of GI effects and slowing gastric emptying. Actually, few studies [82,83,84,85] have investigated the use of GLP1RA in KTRs and their potential interaction with immunosuppressive drugs.

Mahzari et al. [82] investigated the use of semaglutide in 39 KTRs with type 2 DM or PTDM (mean age, 54 ± 9 years; male, 74%; insulin therapy, 85%), demonstrating a reduction in HbA1c and weight during the period of this study. In particular, insulin administration was reduced in 36% of patients. Vigara et al. [86] analyzed 96 diabetic KTRs (mean age, 61.6 ± 9.7 years; male, 56.2%; BMI, 35.8 ± 4.8; PTDM, 43.7%) in therapy with GLP1RA for a period of follow up of 6–12 months. They observed a significant reduction in proteinuria, weight, HbA1c, systolic blood pressure, and total and LDL cholesterol. In particular, they did not report changes in graft function and trough levels of tacrolimus and no cases of acute rejection were registered. The main AEs correlated with the use of GLP1-RA were gastrointestinal AEs, which lea to drug discontinuation in eleven patients.

Other studies [83,84] demonstrated that the use of GLP1RAs in KTRs improved renal outcomes (graft rejection, re-initiation of dialysis, re-transplant), with a decrease in body mass index (BMI) and HbA1c and triglycerides levels. Mahmoud et al. [85] reported data on 1 year of follow up of KTRs with DM comparing treatment with SGLT2is (98 patients), GLP1RAs (41 patients), and standard-of-care medicines (70 patients). All patients were almost 3 months from transplantation. They found that the use of SGLT2is and GLP1RAs is safe in KTRs and induces a significant reduction in HbA1c, body mass index (BMI), and albuminuria.

GLP1RA treatments improve CV outcomes in CKD patients [74,76,77]. Dotan et al. [87] demonstrated that their use in diabetic transplant patients also reduces major cardiovascular events (MACEs) and all-cause mortality (N, 318 patients; mean age, 58.3 ± 11.0 years; male, 69%). Recently, Orandi et al. [88] showed that GLP1RA treatment in KTRs with pre-existing diabetes reduces graft loss and death. This is a large study that used data from the US Renal Data System (USRDS) and compared 1969 GLP1RA-treated patients versus 16,047 GLP1RA-untreated patients (GLP1RA users’ mean age: 57 years vs. 60 years; non-users: female, 39.9% users vs. 35.7% non-users).

In a systematic review and meta-analysis, Krisanapan et al. [89] analyzed nine cohort studies with a total of 338 KTRs, confirming the safety and efficacy of GLP1RA use in transplant patients.

Based on the different mechanisms of action, SGLT2is and GLP1ARs may be used in combination. Juric et al. [90] described a single clinical case of a 31-year-old KT patient that developed PTDM after transplantation. Initially, he started sitagliptin/metformin treatment and subsequently, because of weight gain and poor glycemic control, he started subcutaneous semaglutide and empaglifozin. Treatment was well tolerated, tacrolimus levels remained stable, and reductions in HbA1c and weight loss were observed.

Actually, only two clinical trials are investigating the use of GLP1RAs in KTRs. The HALLAMARK clinical trial (Table 1) [68] is investigating the concomitant use of GLP1RAs with SGLT2is in 20 KTRs with and without diabetes. Patients will receive semaglutide and dapaglifozin for 12 weeks. The aim of the study is to evaluate the effect on salt and water removal, blood pressure, liver stiffness, renal and heart function, and the safety profile.

Sema-RTx [91] is a randomized clinical trial investigating the use of semaglutide in 104 KTRs with post-transplant hyperglycemia. Patients will be randomized 1:1 in semaglutide vs. placebo, both added to the standard of care (usually insulin). The primary objective is to evaluate whether semaglutide is non-inferior to placebo in regulating plasma glucose levels. In addition, the study will investigate the safety profile (in particular, hypoglycemia and immunosuppressive drugs levels) and the effect of semaglutide on graft function, weight, and daily insulin dose.

Overall, studies demonstrate that the use of GLP1ARs in KTRs is effective in controlling blood glucose and reducing weight, with an impact on metabolic balance, and may preserve graft function. The main AEs are gastrointestinal symptoms, which may lead to drug discontinuation without irreversible complications [89,92]. In addition, GLP1RAs may contrast the collateral effects of immunosuppressive drugs such as hyperglycemia and weight gain after transplantation [93].

Furthermore, GLP1RAs may help in losing weight in obese CKD patients to facilitate kidney transplantation. An ongoing study from the university of Canada, OK-TRANSPLANT2 [94], aims to enroll 60 CKD/dialysis patients with obesity (BMI > 35 kg/m^2^). Patients will be randomized in semaglutide subcutaneously once weekly vs. usual care. The GLP1RA group will also receive nutritional and movement advice.

## 6. Nonsteroidal Mineralocorticoid Receptor Antagonist: Finerenone

Finerenone is a novel, selective, ns-MRA that reduces CKD progression and CV risks in DM type 2 CKD patients [27,28,95].

The finerenone in reducing kidney failure and disease progression in diabetic kidney disease (FIDELIO-DKD) [95] and finerenone in reducing CV mortality and morbidity in diabetic kidney disease (FIGARO-DKD) [27] are two placebo-controlled clinical trials that have investigated the use of finerenone in CKD patients. They demonstrate the ability of finerenone in reducing kidney disease progression and improving CV outcomes in patients with mild-to-severe CKD and type 2 DM in treatment with the maximum tolerated dose of a renin–angiotensin system inhibitor (RASi). Based on this evidence, 2022 KDIGO guidelines [29] recommend MRA use in CKD patients with T2DM, eGFR ≥ 25 mL/min/1.73 m^2^, and a normal serum potassium concentration and albuminuria (≥30 mg/g) despite a maximum tolerated dose of RASi.

Finerenone differs from steroidal-MRAs (spironolactone and eplerenone) for its selectivity, potency, and tissue distribution [96]. In particular, finerenone presents a higher selectivity and higher potency compared to steroidal MRAs, leading to less hormone-related effects (gynecomastia, etc.) and an equivalent tissue distribution in the heart and kidney [96].

However, both FIGARO-DKD and FIDELIO-DKD trials did not include KTRs. In these patients, there are concerns about interactions with immunosuppressive drugs, i.e., tacrolimus. Indeed, finerenone [97] presents a first-pass metabolism in the gut wall and liver, and it is metabolized by Cytochrome P450 (CYP) 3A4 (90%) and CYP2C8 (10%) and excreted principally via renal routes (80%). Therefore, finerenone and tacrolimus share the metabolism by CYP3A4. On the other hand, finerenone is not able to affect CYP and drug transporters.

The beneficial effects of finerenone are due to a downregulation of renal proinflammatory/profibrotic genes in response to damage [97]. The use of MRAs in kidney transplant patients have been investigated [98,99,100]. These studies showed that MRAs may prevent the ischemia/reperfusion injury (IRI) in the immediate post-transplant period, and the short- and long-term calcineurin inhibitor nephrotoxicity [98].

Actually, there are no literature data about the use of finerenone in diabetic KTRs, and only one clinical trial is ongoing (EFFEKTOR study). The effect of finerenone in kidney transplantation recipients (EFFEKTOR) study [101] is a multicenter, phase 2 randomized, double-blinded, placebo-controlled clinical trial. KTRs (N 150) will be randomized 2:1 in finerenone to placebo in order to investigate the effect and safety of finerenone in KTRs with particular attention given to kidney and CV events, hyperkaliemia, and AKI. In addition, 50 KTRs will undergo a kidney graft biopsy before and after the active treatment in order to evaluate the mechanisms that may lead to a reduction in interstitial fibrosis progression and CNI toxicity.

## 7. Conclusions

DM is an important comorbidity in KTRs that impacts graft and patient survival. In recent years, new hypoglycemic agents have been approved in CKD patients with improvements in kidney and CV outcomes. Actually, several studies and clinical trials are investigating their use in KTRs. In recent years, the attention for SGLT2i has considerably increased. Much literature data confirm their security and efficacy in KTRs, with an infective risk comparable with non-transplant patients. The beneficial effect of SGLT2i is also related to their effect on lipid metabolism, graft function, erythropoietin production, cognitive impairment [102], and gut microbiota [103].

Based on the gut microbiota correlation with immune response, drug metabolism, and post-transplant complications in KTRs [104] and based on the noted cognitive impairment in CKD patients [105], SGLT2is represent a promising therapeutic option. However, further studies are needed to investigate these positive effects on KTRs. On the contrary, few RCTs are ongoing on the use of GLP1RAs and finerenone in KTRs, with promising data for GLP1RAs and virtually no data for finerenone. In addition to their effect on renal and CV outcomes, GLP1RA may be useful in contrasting hyperglycemia and weight gain in the post-transplant period, reducing the risk of PTDM. Of particular note, finerenone may protect against IRI and reduce calcineurin inhibitor toxicity.

According to the last meeting in Vienna about PTDM, we concluded that more studies are needed to confirm whether these drugs may be used in security and with good effects.

## Figures and Tables

**Table 1:** Diagnostic criteria for diabetes mellitus and prediabetes by the American Diabetes Association (ADA).

Normal Glucose Tolerance	Prediabetes	Diabetes Mellitus
FPG < 100 mg/dL	FPG 100−125 mg/dL	Symptoms of diabetes
2-h PG < 140 mg/dL	2-h PG during 75-g OGTT 7.8−11.0 mmol/L	RPG ≥ 200 mg/dL
HbA1c < 5.7% (< 39 mmol/mol)	HbA1c 5.7−6.4% (39−47 mmol/mol)	FPG ≥ 126 mg/dL
		2-h PG ≥ 200 mg/dL during 75-g OGTT
		HbA1c ≥6.5%(>47 mol/mol)

OGTT: Oral glucose tolerance test (OGTT); HbA1c: hemoglobin A1c; FPG: fasting glucose sample; 2-h PG: 2 h plasma glucose; RPG: random plasma glucose.

**Table 2:** Clinical studies with SGLT2 inhibitors in kidney transplant recipients.

Study	Population	Intervention	Included Criteria	Primary Endpoints	Results
AlKindi et al. [54]	8 KTRS	SGLT2i	T2DM/PTDM Stable renal function	Safety and efficacy	Improvement in HbA1c and body weight Safety
Halden et al. [55]	49 KTRS	Empagliflozin	PTDM Transplanted >1 year Stable renal function Stable immunosuppressive therapy	Safety and efficacy	Improvement in HbA1c and body weight Safety
Mahling et al. [56]	10 KTRs	Empagliflozin	T2DM/PTDM Stable graft function No recurrent UTIs	Efficacy, safety, and effect on allograft function	Stable graft function Reduced HbA1c, body weight, BP Safety
Schwaiger et al. [57]	14 KTRs	Empagliflozin	PTDM Insulin therapy < 40 IU/d HbA1c <8.5% eGFR >30 mL/min/1.72 m^2^ Transplanted > 6 months	Withdrawing insulin	Empagliflozin safe as add-on therapy
Song et al. [58]	50 KTRs	SGLT2i	T2DM/PTDM eGFR > 30 mL/min/1.72 m^2^ None AKI ≤ 30 days None UTIs 6 months	Changes in weight, insulin dosage, HgbA1C, magnesium concentration and safety outcomes	Improvement in weight, hypomagnesemia and insulin usage
Lim et al. [59]	226 KTRs	SGLT2i	T2DM/PTDM SGLT2i treatment > 90 d	Primary composite outcome: all-cause mortality, death-censored graft failure (DCGF), and serum creatinine doubling	Lower risk of primary composite outcome
Sánchez Fructuoso et al. [60]	339 KTRS	SGLT2i	T2DM/PTDM	Safety profile	UTIs 14% Risk factors: prior episode and female sex
Sheu et al. [61]	1970 KTRS	SGLT2i	T2DM/PTDM For SGLT2i users group, administered SGLT2i within the 3 months post-transplant	All-cause mortality MACE MAKE	Reduced all-cause mortality, MACE, MAKE

KTRs = kidney transplant recipients; SGLT2i = Sodium Glucose Co-Transporter Type 2 Inhibitors; T2DM = type 2 diabetes mellitus; PTDM = post-transplant diabetes mellitus; UTIs = urinary tract infections; HbA1c = haemoglobin A1c; eGFR = Estimated Glomerular Filtration Rate; AKI = acute kidney injury; MACE = major adverse cardiac events; MAKE= major adverse kidney events.

## Abbreviations

The following abbreviations are used in this manuscript:

KT	Kidney transplantation
ESKD	End-stage kidney disease
DM	Diabetes mellitus
KDIGO	Kidney Disease: Improving Global Outcomes
KTRs	Kidney transplant recipients
CKD	Chronic kidney disease
NODAT	New-onset diabetes mellitus after transplantation
PTDM	Post-transplantation diabetes mellitus
HbA1c	Hemoglobin A1c
OGTT	Oral glucose tolerance test
IGT	Impaired glucose tolerance
CV	Cardiovascular
ADA	American Diabetes Association
FPG	Fasting glucose sample
2HPG	2 hr plasma glucose
RPG	Random plasma glucose
MM	Mismatching
CNIs	Calcineurin inhibitors
mTORi	Mammalian target of rapamycin inhibitor
MMF	Mycophenolate mofetil
SGLT2is	Sodium glucose co-transporter type 2 inhibitors
GLP1RAs	Glucagon-like peptide-1 receptor agonists
Ns-MRA	Nonsteroidal mineralocorticoid receptor antagonist
HF	Heart failure
FDA	Food and Drug Administration
BMI	Body mass index
AKI	Acute kidney injury
AEs	Adverse events
UTIs	Urinary tract infections
DKA	Diabetic ketoacidosis
eGFR	Estimated glomerular filtration rate
RCT	Randomized clinical trial
MACEs	Major adverse cardiac events
MAKEs	Major adverse kidney events
GIPR	Glucose-dependent insulinotropic polypeptide receptor
TNF-α	Tumor necrosis factor alpha
GI	Gastrointestinal
FAERS	Food and Drug Administration adverse event reporting system
AIN	Acute interstitial nephritis
USRDS	US Renal Data System
RASi	Renin–angiotensin system inhibitor
CYP	Cytochrome
